# Production of ‘Aliança’ papaya seedlings under different dosages and sources of *Lithothamnion* sp

**DOI:** 10.3389/fpls.2025.1630560

**Published:** 2025-08-08

**Authors:** Elmo Pereira Ramos, Diego Borges de Aguiar, Ana Kelly Mota Barbosa, Vinicius de Souza Oliveira, Lúcio de Oliveira Arantes, Edilson Romais Schmildt, Antelmo Ralph Falqueto, Adriano Alves Fernandes, Sara Dousseau-Arantes

**Affiliations:** ^1^ Instituto Capixaba de Pesquisa, Assistência Técnica e Extensão Rural, Linhares, Espírito Santo, Brazil; ^2^ Departamento de Ciências Biológicas, Centro de Ciências Humanas e Naturais, Universidade Federal do Espírito Santo, Avenida Fernando Ferrari, 514, Goiabeiras, Vitória, Espírito Santo, Brazil; ^3^ Departamento de Ciências Agrárias e Biológicas, Centro Universitário Norte do Espírito Santo, Universidade Federal do Espírito Santo, São Mateus, Espírito Santo, Brazil

**Keywords:** *Carica papaya L.*, calcareous algae, nutrition, biostimulant, seedling quality

## Abstract

**Introduction:**

The production ofpapaya seedlings is one of the main steps for establishing crops that present maximum quality performance. Nutritional availability is one of the factors limiting the formation process of quality seedlings, and fertilization is often done without technical criteria. Thus, the objective of this study was to evaluate the effect of different doses of *Lithothamnion* sp., obtained from different sources, on the development and growth of ’Aliança‘ papaya seedlings.

**Methods:**

The study was developed at the Experimental Farm of the Capixaba Institute of Research, Technical Assistance, and Rural Extension, in the municipality of Linhares, north of the state of Espírito Santo. The experimental design used was randomized blocks in a factorial scheme, where the first factor was composed of three commercial sources of *Lithothamnion* sp. (LT Supra®; Algen® and Primaz®). The second factor was composed of six different doses, namely: 0; 2; 4; 6; 8; and 10 kg m^-3^ of *Lithothamnion* sp. At 37 days after sowing, the seedlings were evaluated for the following characteristics: germination percentage; leaf area; stem length; stem mass fraction; root length; stem diameter; leaf dry mass; stem dry mass; root dry mass; and total dry mass. The content of nitrogen, phosphorus, potassium, calcium, magnesium, and sulfur in the leaves and roots was also evaluated.

**Results:**

The use of *Lithothamnion* sp. on ’Aliança‘ papaya seedlings promoted leaf and stem growth and development, dry matter accumulation, and improved germination percentage. *Lithothamnion* sp. from the coast of Espírito Santo (LT supra®) promoted significant gains in germination percentage, leaf area, stem length, root collar diameter, leaf dry matter, stem dry matter, and total dry matter. *Lithothamnion* sp. from the coast of Maranhão (Algen®) and Bahia (Prima®) increased leaf phosphorus and root sulfur levels.

**Discussion:**

*Lithothamnion* sp. promoted a biostimulant effect with improved growth and development in ’Aliança‘ papaya seedlings, with a dosage close to 4 kg m^-3^, with the LT supra® product being the most recommended.

## 1 Introduction

The papaya tree (*Carica papaya* L.) is one of the main tropical fruit species, being distributed across all continents. In Brazil, the species is found in all regions, with the country being responsible for producing approximately 1,107,761 tons of papayas in 2022, with cultivation concentrated in the Northeast and Southeast due to favorable edaphoclimatic conditions. Nationally, the state of Espírito Santo stands out as the main producer, with 426,616 tons of papayas in 2022 (Zucoloto et al., 2023, IBGE - Instituto Brasileiro de Geografia e Estatística, 2022).

Currently, the propagation of papaya in commercial plantations in Brazil is mostly done through seeds, with the production of seedlings being one of the main steps in obtaining crops with plants that express their genetic potential and produce fruits with quantity and quality (Weckner et al., 2016). Among the factors that affect the obtaining of quality seedlings is the availability of nutrients, with the interaction between nutrients and soil affecting the availability of nutrients for plants.

In this sense, the use of compounds that improve seedling nutrition is essential for the success of the crop (Bamaniya et al., 2024). *Lithothamnion* sp. uses calcareous algae and has shown positive effects in improving plant quality as a biostimulant for plants of various species such as jatropha (Evangelista et al., 2016), Arabica coffee (Rodriguez et al., 2017), melon (Negreiros et al., 2019; Marques et al., 2025), tomato (Amatussi et al., 2020), bean (Marques et al., 2023), cucumber (Kakbra, 2024), ornamental plants (Yücedağ and Çiçek, 2024) and potato (Amatussi et al., 2025).

However, for the production of papaya seedlings, nutrition is often done empirically, not respecting technical criteria, which can result in loss of seedling quality and waste of monetary resources, since fertilization can be a significant part of the crop production costs. Furthermore, the mineral composition of algae such as *Lithothamnion* sp. is strongly influenced by the environment (Carvalho et al., 2020). Therefore, the difference in nutrient content due to the location of the extraction site where *Lithothamnion* sp. originates is fundamental to understanding the biostimulant effect (Singh et al., 2025).

Thus, knowledge of seedling production protocols allows for the optimization of resources, reducing environmental and economic impacts. Therefore, studies that effectively identify management methods are essential. Thus, the objective of this study was to evaluate the effect of different doses of *Lithothamnion* sp., obtained from different deposits, on the development of ‘Aliança’ papaya seedlings.

## 2 Materials and methods

**Figure 1:**
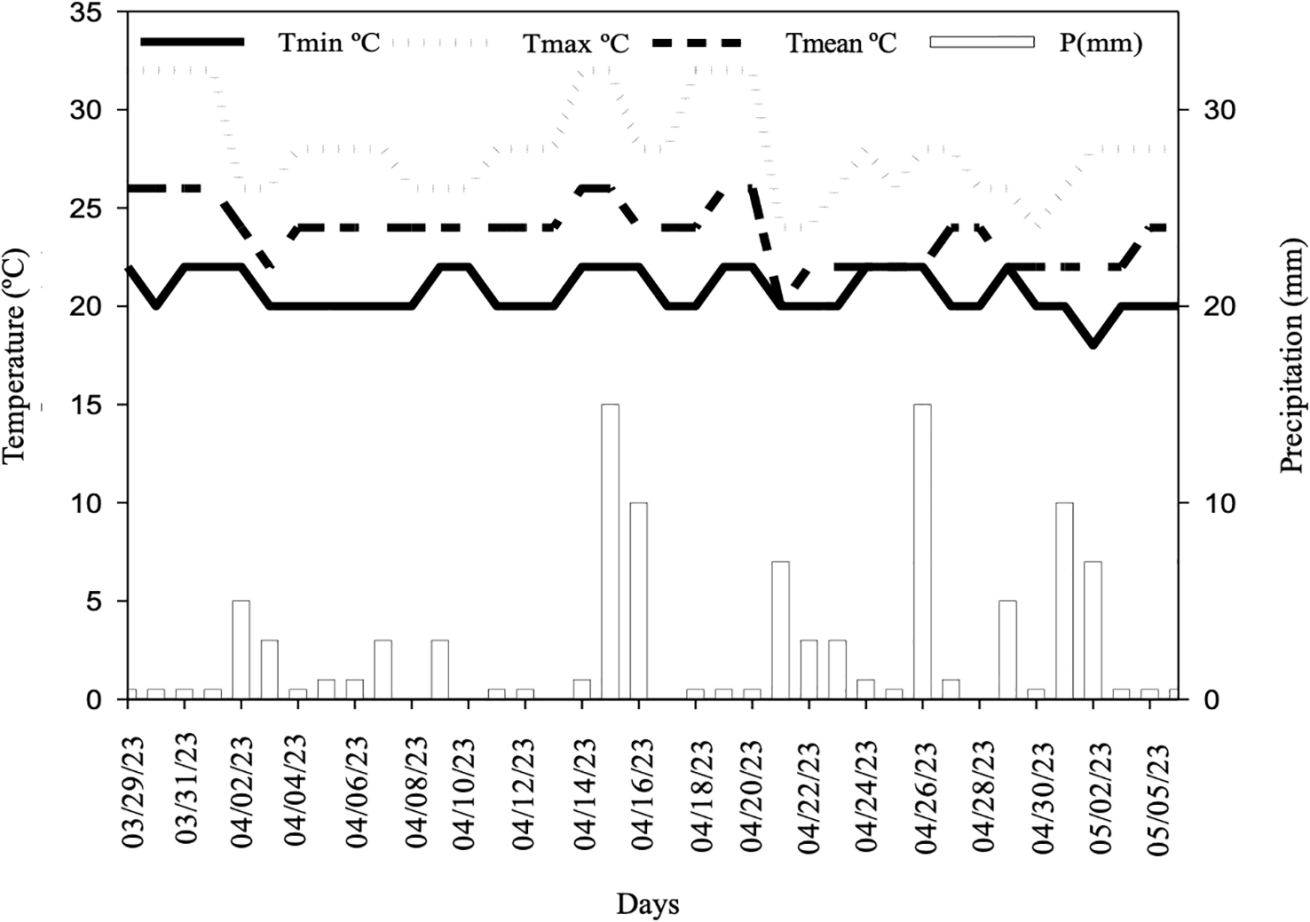
Monthly precipitation (mm) and temperature (°C) covering the period after seed germination until the analysis of seedling quality parameters.

The study was conducted at the Experimental Farm of the Capixaba Institute for Research, Technical Assistance, and Rural Extension, located in the municipality of Linhares in the north of the state of Espírito Santo. The climate of the region, according to the Koppen classification, is tropical Aw, with rain in the summer and dry winters. The precipitation and temperature recorded during the experimental period are shown in **Figure 1**. The experiment was conducted in a greenhouse with a black shade screen with 50% shading and irrigation by micro-sprinklers with a flow rate of 12 L h^-1^ activated by timers every 15 minutes for 10 seconds.

To prepare the seedlings, tubes with a volume of 280 cm^3^ were used, filled with commercial substrate Tropstrato^®^ HT Hortaliças added with 3 g tube^-1^ of Basacote^®^ (3M), in the NPK formulation 18-08-12. Sowing was carried out seven days after filling the tubes with ‘Aliança’ papaya seeds, purchased from a local producer. Three seeds were used per tube, and 10 days after sowing, thinning was carried out, maintaining only one seedling per tube.

The experimental design was randomized blocks in a factorial scheme (3 x 6), where the first factor consisted of three commercial sources of *Lithothamnion* sp., namely: LT Supra^®^; Algen^®^ and Primaz^®^, respectively extracted from deposits located in the states of Espírito Santo, Maranhão and Bahia. Their chemical composition is presented in **Table 1**. The second factor consisted of six different dosages of *Lithothamnion* sp., namely: 0; 2; 4; 6; 8; and 10 kg m^-3^. The treatments were incorporated into the substrate at the time of seedling preparation. Four blocks were evaluated, with 20 seedlings in each block, totaling 1,440 plants in the experimental field.

**Table 1:** Chemical composition of nutrients and humic substances of commercial products of *Lithothamnion* sp. from different extraction sources obtained in the States of Espírito Santo (LT Supra^®^), Maranhão (Algen^®^) and (Bahia Primaz^®^).

Parameter	Unit	Values analyzed		
		Algen^®^	Primaz^®^	LT Supra^®^
Nitrogen (N)	%	0.05	0.05	0.06
Phosphorus (P)		0.06	0.06	0.09
Potassium (K)		0.03	0.04	0.06
Calcium (Ca)		34.28	30.29	31.19
Magnesium (Mg)		3.21	3.62	2.06
Sulfur (S)		0.22	0.28	0.29
Boron (B)	mg kg^-1^	31.17	30.24	48.06
Copper (Cu)		0.16	0.94	0.97
Iron (Fe)		717.01	4527.96	14765.56
Manganese (Mn)		11.91	49.67	481.89
Zinc (Zn)		1.19	2.88	10.50
Sodium (Na)		3744.25	4899.77	8084.48
Fulvic acid	%	6.77	5.40	9.31
Humic acid		3.16	4.91	0.93

Source: Ramos et al. (2023).

At 37 days after sowing, the seedlings were evaluated by the following characteristics: germination percentage (GP), obtained by counting the total number of surviving seedlings and expressed in %; leaf area (LA), measured by the LI-3100 equipment, in cm^2^; stem length (SL), measured with a graduated ruler, from the collar to the apical bud, in cm; stem mass fraction (SMF), obtained by dividing the stem dry mass by the total dry mass of the plant, expressed in g g^-1^, as determined by Poorter et al. (2011); root length (RL), measured with a graduated ruler, in cm, from the base of the collar to the greatest root length; root collar diameter (RCD), with a caliper, in mm; leaf dry mass (LDM), stem dry mass (SDM), root dry mass (RDM) and total dry mass (TDM), measured in g. To obtain LDM, SDM, RDM, and TDM, the plants were dried in an oven with forced air circulation at a temperature of 65°C, and weighed on an analytical balance until they obtained a constant mass.

The accumulation of nitrogen, phosphorus, potassium, calcium, magnesium, and sulfur in the leaves and roots of papaya seedlings was also evaluated, with the measurement expressed in g kg^-1^. To obtain nutritional accumulation, the analyses were performed by the Fullin Laboratory of Agronomic, Environmental Analysis, and Preparation of Chemical Solutions, using the methodology recommended by the Luiz de Queiroz College of Agriculture.

The data were subjected to Pearson correlation analysis and analysis of variance using the F test at 5% probability. The means of the characteristics in relation to the source of origin of *Lithothamnion* sp. were compared by the Tukey test at 5% probability. The effects of *Lithothamnion* sp. dosages on the evaluated characteristics were tested by regression analysis using the F test at 5% probability. When significant, the best model that explained the behavior of each characteristic in relation to the applied dosage was adjusted. The maximum point was obtained by the primary derivative of the regression equations. All analyses and graphical representations were performed with the aid of the Sisvar (Ferreira, 1999) and R (R Core Team, 2024) programs.

## 3 Results

**Table 2** shows the results of the Pearson correlation analysis between the characteristics evaluated. It can be seen that there was a strong correlation, above 0.60, between leaf area and stem length, stem diameter, leaf dry mass, stem dry mass, and total dry mass. Also, a strong correlation was identified between stem length and stem diameter, leaf dry mass, stem dry mass, and total dry mass. Root collar diameter also had a strong correlation with leaf dry mass, stem dry mass, and total dry mass. Likewise, leaf dry mass had a strong correlation with stem dry mass, root dry mass, and total dry mass. Stem dry mass and root dry mass also showed a strong correlation with total dry mass.

**Table 2:** Pearson correlation values ​​for the characteristics germination percentage (GP), leaf area (LA), stem length (SL), stem mass fraction (SMF), root length (RL), root collar diameter (RCD), leaf dry mass (LDM), stem dry mass (SDM), root dry mass (RDM) and total dry mass (TDM), in three different sources of fertilization of papaya seedlings.

Variable	GP	LA	SL	SMF	RL	RCD	LDM	SDM	RDM	TDM
GP	1	0.4715^*^	0.5519^*^	0.3045^*^	0.0417^ns^	0.5312^*^	0.3931^*^	0.4865^*^	0.1233^ns^	0.3727^*^
LA	0.4715^*^	1	0.8531^*^	0.2411^*^	0.5459^*^	0.7502^*^	0.8571^*^	0.8200^*^	0.5069^*^	0.8288^*^
SL	0.5519^*^	0.8531^*^	1	0.3696^*^	0.3618^*^	0.7571^*^	0.7338^*^	0.7860^*^	0.3097^*^	0.6910^*^
SMF	0.3045^*^	0.2411^*^	0.3696^*^	1	-0.1881^ns^	0.4869^*^	0.0428^ns^	0.4827^*^	-0.2890^*^	0.0499^ns^
RL	0.0417^ns^	0.5459^*^	0.3618^*^	-0.1881^ns^	1	0.1150^ns^	0.3846^*^	0.2149^*^	0.3675^*^	0.3787^*^
RCD	0.5312^*^	0.7502^*^	0.7571^*^	0.4869^*^	0.1150^ns^	1	0.7100^*^	0.8125^*^	0.2822^*^	0.6757^*^
LDM	0.3931^*^	0.8571^*^	0.7338^*^	0.0428^ns^	0.3846^*^	0.7100^*^	1	0.8530^*^	0.6705^*^	0.9666^*^
SDM	0.4865^*^	0.8200^*^	0.7860^*^	0.4827^*^	0.2149^*^	0.8125^*^	0.8530^*^	1	0.5443^*^	0.8818^*^
RDM	0.1233^ns^	0.5069^*^	0.3097^*^	-0.2890^*^	0.3675^*^	0.2822^*^	0.6705^*^	0.5443^*^	1	0.8136^*^
TDM	0.3727^*^	0.8288^*^	0.6910^*^	0.0499^ns^	0.3787^*^	0.6757^*^	0.9666^*^	0.8818^*^	0.8136^*^	1

^*^Significant at 5% probability of error, by T-test.

After the analysis of variance, no significant interaction was found between the factors of commercial sources of *Lithothamnion* sp. and doses applied by the F test at 5% probability. **Table 3** shows the comparison of the means of the growth and phytomass characteristics of the seedlings in relation to the fertilizer source used. It can be seen that for the characteristics stem mass fraction (SMF), root length (RL), and root dry mass (RDM), there were no statistically significant differences between the fertilizer sources. For the other variables, the seedling production achieved with the use of LT Supra^®^ was superior to the other treatments.

**Table 3:** Mean values ​​of germination percentage (GP),leaf area (LA), stem length (SL), stem mass fraction (SMF), root length (RL), root collar diameter (RCD), leaf dry mass (LDM), stem dry mass (SDM), root dry mass (RDM) and total dry mass (TDM), in three different sources of fertilization of papaya seedlings.

Fonte	GP	LA	SL	SMF	RL	RCD	LDM	SDM	RDM	TDM
Primaz^®^	71.25b	38.04b	8.36b	0.225a	10.66a	2.39b	0.077b	0.030b	0.024a	0.133b
Algen^®^	75.83b	35.44b	7.84b	0.214a	10.29a	2.34b	0.072b	0.026b	0.022a	0.121b
LT Supra^®^	85.62a	44.95a	9.54a	0.236a	10.43a	2.73a	0.093a	0.037a	0.027a	0.157a

Averages followed by the same letter between columns do not differ from each other by the Tukey test at 5% probability.

**Figure 2:**
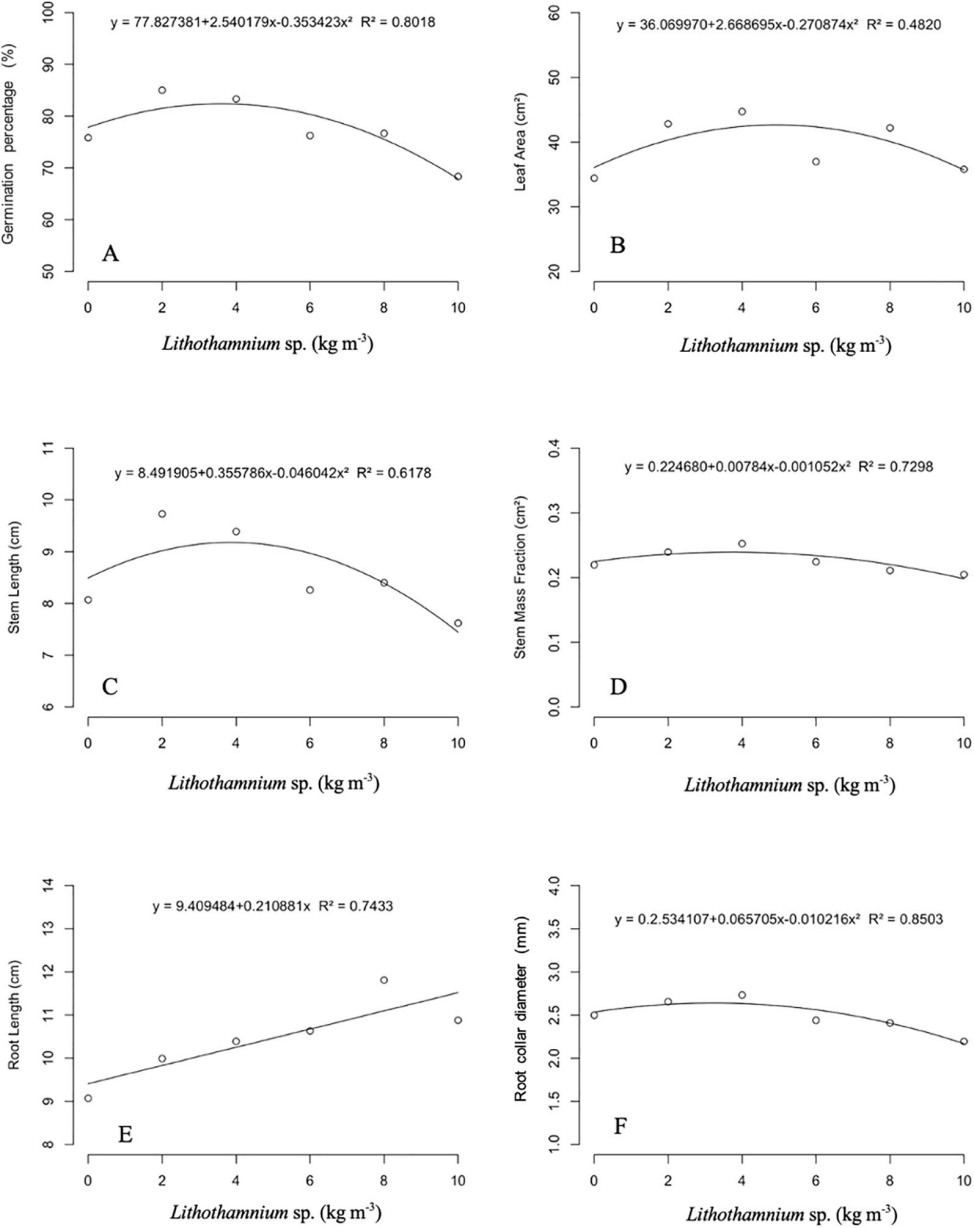
Effect of different doses of *Lithothamnion* sp. on germination percentage **(A)**, leaf area **(B)**, stem length **(C)**, stem mass fraction **(D)**, root length **(E)**, root collar diameter **(F)** of ‘Aliança’ papaya seedlings.

The behavior of seedling growth characteristics in relation to the applied dosage of *Lithothamnion* sp. is represented in **Figure 2**. The germination percentage index presented a quadratic fit, with a maximum point of 82.40% at the dosage of 3.59 kg m^-3^ and coefficient of determination (R^2^) of 0.8018 (**Figure 2A**). The leaf area presented a quadratic effect, with R^2^ of 0.4820 and a maximum point of 42.64 cm^2^ at the dosage of 4.92 kg m^-3^ (**Figure 2B**). For the stem length, the fit observed was quadratic with R2 of 0.6178 and a maximum point of 9.18 cm at the dosage of 3.86 kg m^-3^ (**Figure 2C**). The stem mass fraction presented a quadratic fit with a maximum point of 0.239 g at a dosage of 3.72 kg m^-3^ and R^2^ of 0.7298 (**Figure 2D**). The fit found for root length was linear of the first degree increasing as the dosage increased, with R^2^ of 0.7433 (**Figure 2E**). The root collar diameter had a quadratic behavior and a maximum point of 2.64 mm at a dosage of 3.21 kg m^-3^, with R^2^ of 0.8503 (**Figure 2F**).

**Figure 3:**
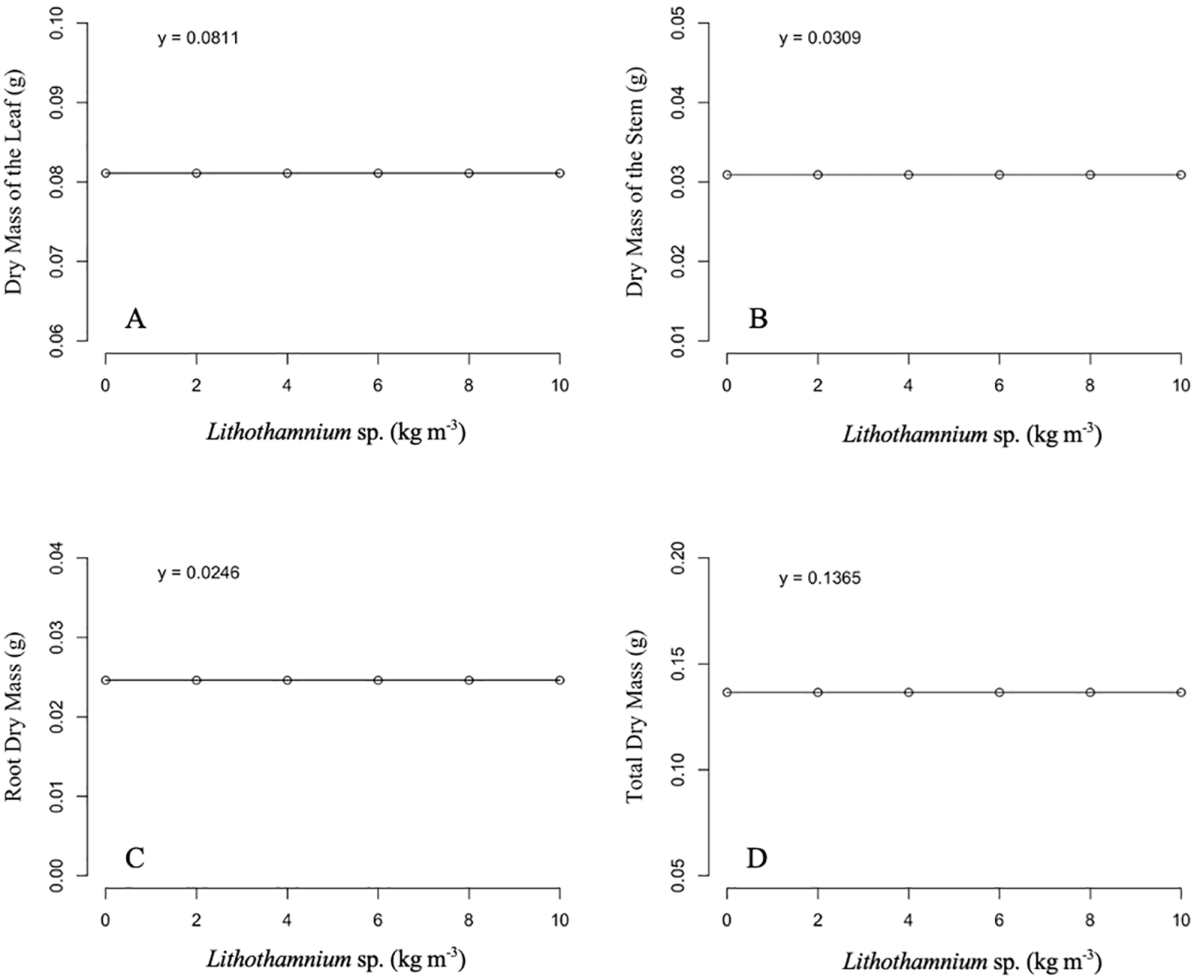
Effect of different doses of *Lithothamnion* sp. on leaf dry mass **(A)**, stem dry mass **(B)**, root dry mass **(C)** and total dry mass **(D)** of ‘Aliança’ papaya seedlings.

For the characteristics related to phytomass, no statistical differences were identified between the applied dosages (**Figure 3**). Thus, for the leaf dry mass, stem dry mass, root dry mass, and total dry mass, the ‘Aliança’ papaya seedlings presented respective averages of 0.0811, 0.0309, 0.0246, and 0.1365 g in all *Lithothamnion* sp. dosages that were submitted.

Regarding nutrient accumulation (**Table 4**), regardless of the source from which *Lithothamnion* sp. was extracted, only the sulfur concentration in the leaves of the Primaz^®^ and Algen^®^ treatments was significantly higher than that of LT Supra^®^. For the other nutrients evaluated in the leaves, the quantity was significantly equal, even though the nutritional composition of each source was different. For accumulation in the roots, the Primaz^®^ and Algen^®^ treatments were statistically superior to the LT Supra^®^ treatment for phosphorus accumulation. Potassium had greater accumulation in the roots of the Algen^®^ treatment. Magnesium showed greater accumulation in the Primaz^®^ and LT Supra^®^ treatments. The accumulation of nitrogen, calcium, and sulfur did not show significant differences between the treatments.

**Table 4:** Average values ​​of nitrogen (N), phosphorus (P), potassium (K), calcium (Ca), magnesium (Mg) and sulfur (S) accumulation in leaves and roots of ‘Aliança’ papaya seedlings using *Lithothamnion* sp. extracted from different sources.

Sources	N		P		K		Ca		Mg		S	
	Leaves	Roots	Leaves	Roots	Leaves	Roots	Leaves	Roots	Leaves	Roots	Leaves	Roots
Primaz^®^	57.42a	2.86a	2.73a	12.25a	27.14a	40.05b	12.94a	11.94a	9.07a	5.25a	6.98a	6.82a
Algen^®^	56.51a	3.13a	2.66a	11.95a	27.24a	46.46a	13.20a	11.46a	9.62a	4.44b	6.85a	7.40a
LT Supra^®^	55.72a	3.11a	2.66a	11.08b	27.76a	38.33b	13.09a	11.97a	9.49a	5.34a	6.18b	6.13a

Averages followed by the same letter between columns do not differ from each other by the Tukey test at 5% probability.

**Figure 4:**
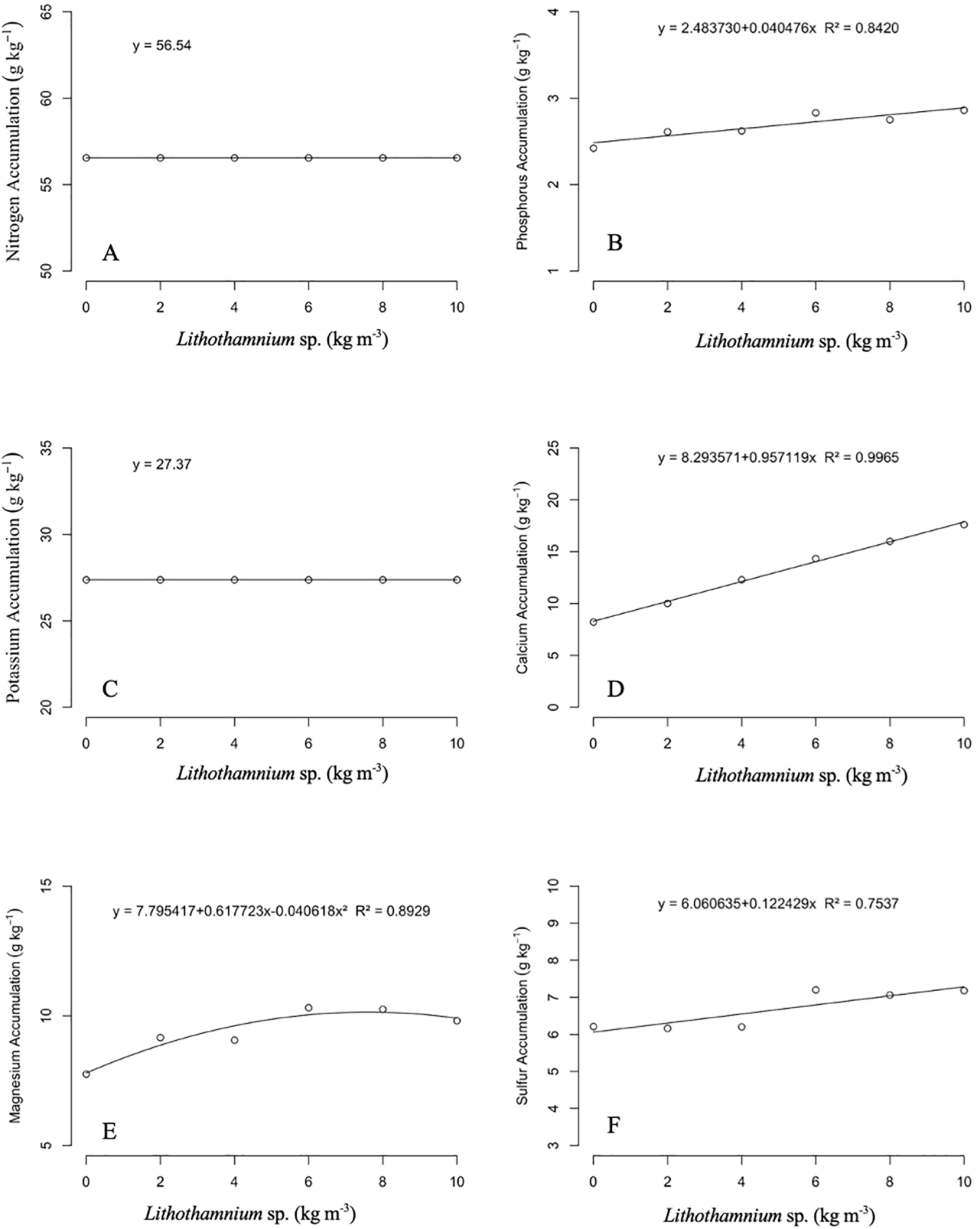
Effect of different doses of *Lithothamnion* sp. on the accumulation of nitrogen **(A)**, phosphorus **(B)**, potassium **(C)**, calcium **(D)**, magnesium **(E)** and sulfur **(F)** in leaves of ‘Aliança’ papaya seedlings.

**Figure 5:**
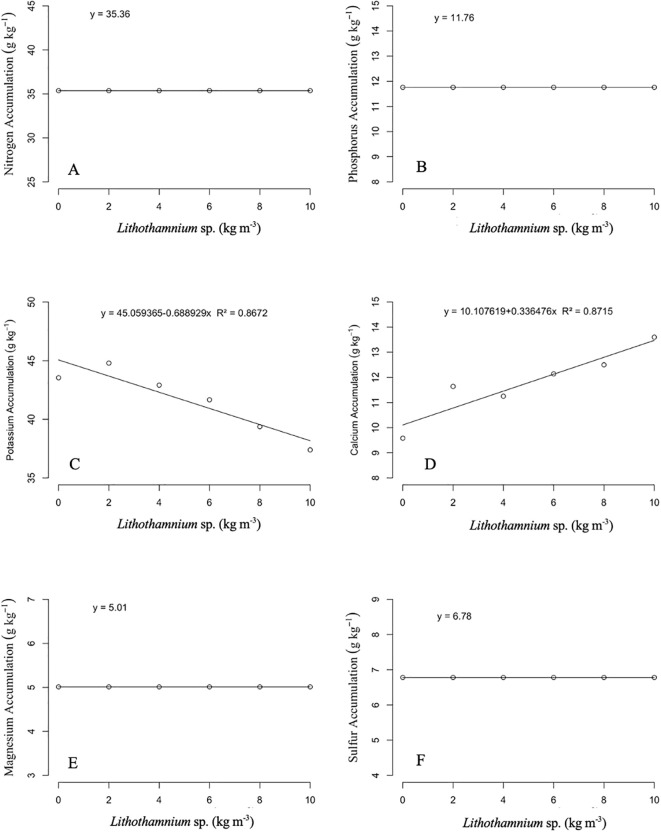
Effect of different doses of *Lithothamnion* sp. on the accumulation of nitrogen **(A)**, phosphorus **(B)**, potassium **(C)**, calcium **(D)**, magnesium **(E)** and sulfur **(F)** in roots of ‘Aliança’ papaya seedlings.

The accumulation of macronutrients in the leaves of ‘Alianca’ papaya seedlings in relation to the dosage of *Lithothamnion* sp. applied can be seen in **Figure 4**. The accumulation of nitrogen and potassium did not present differences between the dosages used, with averages of 56.54 and 27.37 g kg^−^¹, respectively. For the accumulation of phosphorus, calcium, and sulfur, the behavior observed was linear of the first degree, increasing with R² of 0.8420, 0.9965, and 0.7537, respectively. The accumulation of magnesium had a quadratic fit, with the greatest accumulation of 10.14 g kg^−^¹ in the dosages of 7.60 kg m^−^³ of *Lithothamnion* sp. and R² of 0.8929. For the accumulation of macronutrients in the roots of ‘Alianca’ papaya seedlings as a function of the dosage of *Lithothamnion* sp. applied (**Figure 5**). The nutrients nitrogen, phosphorus, magnesium, and sulfur did not present significant differences between treatments, with respective averages of 35.36, 11.76, 5.01, and 6.78 g kg^-1^. The accumulation of potassium in the roots had a decreasing first-degree linear effect with an R² of 0.8672. The accumulation of calcium had an increasing first-degree linear behavior and an R² of 0.8715.

## 4 Discussion

*Lithothamnion* sp. originating from the coast of Espírito Santo (LT supra^®^) differs from the others due to its high levels of micronutrients (B, Fe, Mn, Na, and Zn). This difference, associated with the high levels of calcium and magnesium carbonate common to *Lithothamnion* sp., positively influenced the nutrition of ‘Aliança’ papaya seedlings, as demonstrated by the higher averages of the LT Supra^®^ source. While micronutrients in high concentrations are harmful to plants, on the other hand, they are essential for their growth and development when in low concentrations (Sidhu et al., 2019).

These nutrients play important roles in plant metabolism that may explain the results obtained for the LT Supra^®^ source. Boric acid acts on cell structure, cell division and elongation, regulation of ion absorption in the cell membrane, plant-water relationship, photosynthesis, starch metabolism, nitrogen fixation and metabolism, abiotic stress management, and signaling with gene transcription factors (Kohli et al., 2023). Fe^2+^ acts on chlorophyll synthesis and photosynthesis as a structural part of the ferredoxin and ferredoxin-NADP+-reductase proteins in the electron transport chain (Taiz et al., 2017). It also acts on respiration and metabolism of the nitrogen element and other products (Ning et al., 2023). Mn²^+^ acts on photosynthesis, respiration, elimination of reactive oxygen species, defense against pathogens, and hormonal signaling (Alejandro et al., 2020). Zn^2+^ promotes starch formation and seedling vigor (Sidhu et al., 2019) and, therefore, greater plant growth and development.

Furthermore, the composition of *Lithothamnion* sp. presents humic substances that, according to Jindo et al. (2020), exert a biostimulant function in plants. In papaya seedlings, the use of humic substances promoted growth (Dias et al., 2020) due to increased nutrient absorption and stimulation of auxin production, which in turn plays a fundamental role in root development (Jindo et al., 2020). Thus, the greater availability of nutrients combined with the biostimulation of humic substances may have favored the results observed for the LT Supra^®^ source.

It is noted that, in general, the characteristics related to seedling growth were directly affected by the dosage of *Lithothamnion* sp. applied, with the exception of root length, which showed increasing average values ​​as the dosage increased. For the other characteristics, both the lack and excess of *Lithothamnion* sp. available for the ‘Aliança’ papaya seedlings may have generated a stressful situation for the plants, resulting in a negative effect on the characteristics analyzed, resulting in reduced seedling quality.

Under stressful conditions, one of the first adaptations of plants is the reduction of leaf area (Taiz et al., 2017). Leaves are the main organs responsible for photosynthesis in plants. Therefore, the reduction of the photosynthetically active area of ​​the plant influences the accumulation of carbohydrates that are essential for the formation of structures and maintenance of biochemical processes. Thus, a larger leaf area significantly interferes with plant development, allowing for higher seedling quality (Melo et al., 2007). This fact can be proven due to the high correlation between leaf area, stem length, stem diameter, and dry mass accumulation, which occurs because carbohydrate production directly influences growth characteristics.

It is also observed that, in our studies, the dosage of *Lithothamnion* sp. that presented the highest germination percentage (3.59 kg m^−^³) in papaya seedlings is close to the dosages that provided the highest values ​​of stem length (3.86 kg m^−^³), stem mass fraction (3.72 kg m^−^³), and root collar diameter (3.21 kg m^−^³). This association can be explained because plants with greater height and root collar diameter are more robust, which makes them more difficult to damage, facilitating their development and ensuring greater survival (Melo et al., 2007). Furthermore, both root collar diameter and plant height are characteristics that are easily measured, and the values ​​can be easily obtained without specific equipment; thus, these characteristics are used by many nurseries to transplant seedlings to the field (Dassie et al., 2017).

Thus, in summary, it is possible to conclude that *Lithothamnion* sp. from the State of Espírito Santo, in the form of the commercial product LT supra^®^, proved to be more suitable, allowing better development of papaya seedlings, as evidenced by the higher average values ​​of germination percentage, leaf area, stem length, stem diameter, leaf dry mass, and stem dry mass. Likewise, doses of *Lithothamnion* sp. close to 4.00 kg m^−^³ were responsible for providing the seedlings with better morphological development and germination percentage.

Because they have a high content of calcium and magnesium carbonate in their composition, *Lithothamnion* sp., when made available via the root, raises the pH of the substrate, which can have a direct effect on the availability of nutrients for plants (Ramos et al., 2023; Silva et al., 2023). Under high pH conditions, the macronutrients N, P, K, Ca, Mg, and S become more available to plants. On the other hand, high pH makes the micronutrients Fe, Cu, Mn, Zn, and B unavailable to plants (Gondal et al., 2021). Specifically for papaya seedlings, it is observed that the macronutrients in the sequence of K, Ca, Mg, S, and P and the micronutrients Fe, Zn, Mn, Ni, and Cu are the most required by the crop (Souza et al., 2021).

However, it should be noted that the development of papaya seedlings does not depend solely on one nutrient in isolation, with the nutritional balance being responsible for full growth. Thus, despite the increasing calcium levels observed as a result of the increase in doses of *Lithothamnion* sp. in the leaves and roots of papaya seedlings, the use of this substance should be done with caution, since Ca²^+^ (the form in which calcium is absorbed), in high concentrations, interferes with the absorption of other nutrients, either in synergism or antagonism, that are reflected in the growth and development of plants (Weng et al., 2022).

Ca^2+^ is a nutrient in high demand by plants because it is a structural component and an important messenger in a wide range of physiological aspects of development, such as nutritional aspects and those related to biotic and abiotic stresses (Thor, 2019). As a messenger in plant mineral nutrition, it provides signals for the absorption of macronutrients N, P, K, and Mg and micronutrients Fe and Mn (Wang et al., 2023). Calcium signaling is induced by the level of Ca^2+^ in the cytoplasm (Srivastava et al., 2020; Wang et al., 2023), which also signals the absorption and homeostasis of Ca^2+^ itself (Wang et al., 2023).

It is important to mention that potassium accumulation in the roots decreased linearly as a function of *Lithothamnion* sp. doses. The reduction in potassium accumulation may be related to the nutritional imbalance between calcium and potassium. These two nutrients are cationic with similar absorption sites. This characteristic presents antagonism as the supply of Ca²^+^ increases (Weng et al., 2022). This occurs because both calcium and potassium are absorbed by plants in the cationic forms Ca²^+^ and K^+^, respectively (Fernandes et al., 2018). Thus, the ions are transported by the same mechanism of the cell plasma membrane, with P3A-type H-ATPases, which are capable of transporting Ca^2+^, K^+^, Na^+^, and Zn^2+^, which can lead to antagonism and competitive inhibition between these nutrients (Rietra et al., 2017). Furthermore, the K^+^ uptake pathway, the CBL/CIPK23 complex (Calcineurin B-like proteins/CBL-interacting protein kinases), is regulated by Ca^2+^ (Tang et al., 2020).

Therefore, high concentrations of calcium can significantly interfere with the dynamics of potassium absorption. This antagonism was evidenced in the application of *Lithothamnion* sp. at a high dose in carrots (*Daucus carota* L.), which resulted in losses in K^+^ absorption and, consequently, in productivity (Rodrigues Neto et al., 2021). However, it should be noted that although potassium is the nutrient most required by papaya crops, its greatest need occurs at the fruit development stage, between 150 and 270 days after planting, which does not interfere with seedling development (Coelho Filho et al., 2007; Zucoloto et al., 2023).

Magnesium, in the form absorbed by plants as Mg²^+^, is another essential nutrient for plant development, being present in the chlorophyll molecule, nucleic acids, and proteins, in addition to being an important enzymatic cofactor (Wang et al., 2023). Its balanced supply in the initial phase of development positively affects subsequent phases in the proportion of aerial part-root biomass (Hauer-Jákli and Tränkner, 2019).

Mg^2+^ accumulation in roots is influenced by greater availability in the substrate and, consequently, by plant absorption due to increased substrate pH as an effect of *Lithothamnion* sp., given its composition rich in calcium carbonate and magnesium (Ramos et al., 2023). Thus, it is affected by a greater presence of Ca^2+^, which also acts as a nutritional messenger playing a critical role in regulating the dynamic homeostasis of Mg^2+^ (Wang et al., 2023). Ca^2+^ signaling through CBLs (calcineurin B-like proteins) stores Mg^2+^ in the vacuole to balance amounts in the cytosol (Tang et al., 2020). This evidence indicates the importance of Ca^2+^ in plant biostimulation with the application of *Lithothamnion* sp.

As in the roots, calcium accumulation in the leaves was linearly increasing, indicating the possibility of applying higher doses of *Lithothamnion* sp. in ‘Aliança’ papaya seedlings. These results point to the nutritional demand for Ca^2+^ in the papaya tree, corroborated by Souza et al. (2021), who found calcium to be the second most accumulated mineral. Nutritional demand, although not the focus of the study, interferes with the dynamics of nutrient absorption and accumulation.

Furthermore, the accumulation of magnesium in the leaves indicates greater sensitivity of papaya to high concentrations of this nutrient, since increasing the doses of *Lithothamnion* sp. resulted in a slight inhibition of accumulation from the adjusted dose of 7.60 kg m^-3^. This result also demonstrates the antagonism of Ca^2+^ in the absorption of Mg^2+^, corroborated by Madani et al. (2015) in a study with papaya that, when increasing the doses of calcium, found a decrease in the accumulation of Mg^2+^ in the leaves. This occurs because, as previously mentioned for potassium, magnesium is a cationic ion, like calcium, and there is competition for the absorption mechanisms in the cell membrane. Therefore, the imbalance of these nutrients affects the absorption of other cations because they compete for the same absorption sites (Bang et al., 2021). For example, K^+^ has a high sensitivity in absorption when in a medium with high concentrations of Ca^2+^ and Mg^2+^ (Wacal et al., 2019). Mg²^+^ is an essential nutrient for plant growth and development; its adequate supply allows an increase in the net assimilation of CO_2_, balance in the partition of biomass between the aerial part and the root, and a reduction in reactive oxygen species (Hauer-Jákli and Tränkner, 2019).

Despite the robustness of the data found in this study, the experimental conduction was carried out in a single location and during a single production cycle. Therefore, it is recommended to carry out complementary studies during other production cycles, with the addition of other cultivars, at other times, and in other cultivation environments, in order to validate the data obtained in this study. We therefore suggest conducting field tests, considering that we obtained positive results in the development, growth, and germination percentage of ‘Aliança’ papaya seedlings with 4 kg m^-3^ of *Lithothamnion* sp.

## 5 Conclusion

*Lithothamnion* sp. showed a biostimulant effect on papaya seedlings by promoting the growth and development of leaves and stems, in addition to the accumulation of dry mass, as well as the germination percentage.

The doses of *Lithothamnion* sp. influenced the growth and development of papaya seedlings. The dose that best reflected the quality of ‘Aliança’ papaya seedlings was close to 4 kg m^-3^.

The difference in the composition of *Lithothamnion* sp. originating from the coast of Espírito Santo (LT supra^®^) was more efficient in promoting biostimulant effects on the growth and development of ‘Aliança’ papaya seedlings.

*Lithothamnion* sp. from the coast of Maranhão (Algen^®^) and Bahia (Primaz^®^) were more efficient in nutrient accumulation.

## Data Availability

The original contributions presented in the study are included in the article/supplementary material. further inquiries can be directed to the corresponding author/s.
